# *Tetrastigma hemsleyanum* Vine Flavone Ameliorates Glutamic Acid-Induced Neurotoxicity via MAPK Pathways

**DOI:** 10.1155/2020/7509612

**Published:** 2020-03-22

**Authors:** Qiang Chu, Yonglu Li, Zheng Hua, Yaxuan Wang, Xin Yu, Ruoyi Jia, Wen Chen, Xiaodong Zheng

**Affiliations:** ^1^State Key Laboratory of Silicon Materials, School of Materials Science and Engineering, Zhejiang University, Hangzhou 310027, China; ^2^Department of Food Science and Nutrition, National Engineering Laboratory of Intelligent Food Technology and Equipment, National-Local Joint Engineering Laboratory of Intelligent Food Technology and Equipment, Key Laboratory for Agro-Products Postharvest Handling of Ministry of Agriculture, Zhejiang Key Laboratory for Agro-food Processing, Fuli Institute of Food Science, Zhejiang University, Hangzhou 310058, China

## Abstract

Glutamic acid (Glu) is a worldwide flavor enhancer with various positive effects. However, Glu-induced neurotoxicity has been reported less. *Tetrastigma hemsleyanum* (TH), a rare herbal plant in China, possesses high medicinal value. More studies paid attention to tuber of TH whereas vine part (THV) attracts fewer focus. In this study, we extracted and purified flavones from THV (THVF), and UPLC-TOF/MS showed THVF was consisted of 3-caffeoylquinic acid, 5-caffeoylquinic acid, quercetin-3-O-rutinoside, and kaempferol-3-O-rutinoside. *In vitro*, Glu caused severe cytotoxicity, genotoxicity, mitochondrial dysfunction, and oxidative damage to rat phaeochromocytoma (PC12) cells. Conversely, THVF attenuated Glu-induced toxicity via MAPK pathways. *In vivo*, the neurotoxicity triggered by Glu restrained the athletic ability in *Caenorhabditis elegans* (*C. elegans*). The treatment of THVF reversed the situation induced by Glu. In a word, Glu could cause neurotoxicity and THVF owns potential neuroprotective effects both *in vitro* and *in vivo* via MAPK pathways.

## 1. Introduction

Glutamic acid (Glu), as one of the basic amino acids, is widely applied in industry. Glu is involved in many crucial chemical reactions in the body and plays an important role in protein metabolism in organisms [1]. Meanwhile, it is ubiquitous in the human diet; as a flavor enhancer and food additive in food field, Glu is wildly used to improve the taste of beverages and foods and preserves the freshness of animal food [2, 3]. For example, sodium glutamate, commonly known as monosodium glutamate, is a typical flavor agent that can be used alone or with other amino acids [4]. In addition, studies have proven that Glu is an excellent hair-generating agent. Ottersen et al. reported that Glu effectively promoted the proliferation of hair papilla cells; besides, it can also expand blood vessels and accelerated blood circulation, resulting in hair regeneration [5, 6]. It has also been estimated that Glu poses the ability to reduce wrinkles [7]. As an auxiliary drug for liver diseases, Glu is taken and combined with blood ammonia to form glutamine, which can relieve the toxic effect of ammonia in the metabolic process, thus preventing and treating hepatic coma [8, 9]. Besides, brain tissue cannot oxidize amino acids except glutamate, and therefore, glutamine can be used as an energy substance to improve the function of the brain [10, 11]. As a supplement to the nerve center and cerebral cortex, Glu exhibits a certain effect on the treatment of concussion, nerve damage, epilepsy, and mental retardation [12, 13]. Data show that Glu peaks the largest productive amino acid variety worldwide.

Although Glu shows an irreplaceable role in various fields, it may also turn from a protective agent to a neurotoxin in many cases. A growing number of studies have found that the Glu is closely associated with the etiology and pathology of many neurological and psychiatric disorders, such as cerebral ischemia, epilepsy, Alzheimer's disease, Huntington's disease, schizophrenia, and Pico disease [14]. Oxidative stress caused by Glu is an important cause of neurodegenerative diseases [15, 16], high concentration of Glu inhibits cysteine/glutamate antiporter or system x-CT in neuronal cells, causes calcium overload that interferes with mitochondrial respiratory chain function, thereby inhibiting cystine uptake and causing intracellular glutathione (GSH) deprivation and ROS accumulation, and ultimately leading to cell necrosis or apoptosis [17, 18]. Moreover, studies have shown that the caspase-dependent apoptotic pathway is related with Glu-neurotoxicity [19].

Natural plant extracts are attracting researchers' attention because of their safety, nontoxicity, and various biological activities [20]. Many natural plants have been already proved to exhibit various bioactive capacities such as antioxidative activity, anti-inflammatory activity, hypolipidemic effects, and even antitumor capacity [20]. *Tetrastigma hemsleyanum* Diels et. Gilg (TH), initially used for folk treatment of cancer (Li et al., 2019), is now not only a traditional Chinese medicine but also a type of functional food. Previous studies have shown that TH has antioxidant, anti-inflammatory, anticancer, and immunomodulatory properties that can effectively treat high fever, infantile febrile seizures, pneumonia, asthma, hepatitis, rheumatism, menstrual disorders, sore throats, and sores [21, 22]. It has reported that TH contains many phytochemicals, such as flavonoids, phenolic acids, polysaccharides, and phytosterols, resulting in its various biologically activities such as anti-inflammatory, antioxidant, antiproliferative, antitumor, and antiviral effects [22–24]. However, there are fewer studies that paid attention to vine of TH, which is usually regarded useless and often discarded as a by-product, resulting in a waste of source.

In this study, we have extracted and purified the THVF, then identified and characterized the main compounds of THVF by UPLC-TOF/MS. In addition, we adopted the PC12 cell line and evaluated the protective effects of the THVF against damage induced by Glu *in vitro*. Meanwhile, western blot assay was conducted to unearth the underlying mechanism and the possible signal pathways involved in the protective effects. Furthermore, we assessed THVF's possible protective effects for *Caenorhabditis elegans* (*C. elegans*) against Glu-induced injury and its potential function in nematode physiological activity.

## 2. Materials and Methods

### 2.1. Extraction and Purification of THVF

*T. hemsleyanum* vines were first washed, dried, smashed into powder, and extracted with 80% ethanol at 45°C for 90 min by ultrasonication (with the ratio of material and solution in 1 : 5). The above extraction produce was repeated three times. Then, the filtered fluid was collected and settled at 4°C overnight. On the second day, the filtrate was centrifuged at 4000 r/min for 10 min to gain the supernatants. Then, the supernatants were evaporated to concentrate under reduced pressure at 45°C, the concentrations were later centrifuged at 10000 r/min, and then the supernatants were loaded onto an equilibrated AB-8macroporous resin column (Ø 3.2 × 60 cm) for further purification. Finally, the eluted fluid was evaporated and lyophilized and later stored at -80°C for further research.

### 2.2. Identification of THVF

An Ultra Performance Liquid Chromatography (UPLC) system (Waters, Milford, MA, USA) equipped with a triple-Time-of-Flight mass spectrometry (TOF/MS) system (AB SCIEX, Triple TOF 5600+, Framingham, MA, USA) on a Promosil C18 column (4.6 mm × 250 mm, 5 *μ*m) was used to identify flavonoid compounds. The ingredients of the mobile phase are acetonitrile (A) and 0.1% aqueous formic acid (B). The linear gradient of phase B was 0−1 min (95%), 1−21 min (95–85%), 21−46 min (85–75%), 46–56 min (75–95%), and 56−60 min (95%). The flow rate was 0.8 mL/min, and the injection amount was 5 *μ*L. The mass spectrometry was operated in a negative ion mode at a temperature of 550°C, and the source voltage was 4.5 kV. Ions were recorded from *m*/*z* 100–1500, and the wavelength for the ultraviolet (UV) detector was set as 280 nm.

### 2.3. Cell Culture and Treatments

PC12 cell line was obtained from Shanghai Institute of Cell Biology (Shanghai, China) and cultured in Dulbecco's modified Eagle's medium (DMEM) supplemented with 10% fetal bovine serum (FBS) and 1% penicillin-streptomycin solution in an incubator with 5% CO_2_ at 37°C. THVF powder was always freshly dissolved in DMEM with 10% FBS before use. After being cultured for 24 h, the cells were washed with phosphate-buffered saline (PBS) twice and then pretreated with different concentrations of THVF for another 24 hours. Later, Glu (20 mM) was added for 24 h in the absence/presence of different concentrations of THVF.

### 2.4. Cell Viability Assays

The assays of cell viability were carried out according to our previous protocol [21]. PC12 cells were seeded onto a 96-well plate, and 3-(4,5-dimethyl-2-thiazolyl)-2,5-diphenyl-2-H-tetrazolium bromide (MTT) diluted with serum-free DMEM at a final concentration of 0.5 mg/mL was added to each well after different treatments. After 4 h of incubation at 37°C, the formazan precipitate was dissolved in 150 *μ*L of dimethyl sulfoxide (DMSO) and shook for 15 minutes and the absorbance was measured at 570 nm with a spectrophotometer. The viability of the untreated group was regarded as 100%, and each experiment was repeated at least three times.

### 2.5. Fluorescent Probes Staining for PC12 Cells

After cell treatment, serum-free DMEM, respectively, containing 6 kinds of fluorescent probes were applied, including 2,7-dichlorofluorescein diacetate (DCFH-DA), DHE, aphthalene-2,3-dicarboxaldehyde (NDA), Rhodamine 123, 10-N nonyl acridine orange (NAO), and Hoechst 33258. Cells were washed twice in PBS buffer after treatments and incubated in free-serum DMEM containing the probe at 37°C for 30 min. Then, cells were washed three times with PBS buffer and detected on a fluorescence microscope (Nikon) with different filters at identical acquisition settings. Image-Pro Plus 6.0 software was adopted to analyze the densitometry.

### 2.6. Determination of the Level of SOD, MDA, and GSH

Cells were treated with different treatments, after washing with PBS buffer twice, 500 *μ*L WB/IP cell lysis buffer was added, and cells were scratched and collected. An ultrasonic shredder and centrifuge (Sigma, USA) were used, and the supernatants were collected for further assay. SOD, MDA, and GSH contents were determined with assay kits purchased from Beyotime Biotechnology (Jiangsu, China).

### 2.7. Western Blot

Total protein of cells was prepared using the WB/IP lysis buffer (Beyotime Biotechnology, Jiangsu, China). Equal amounts of protein were subjected to sodium dodecyl sulfate-polyacrylamide gel electrophoresis (SDS-PAGE) and transferred to polyvinylidene fluoride (PVDF) membranes. Membranes were probed with primary antibodies, and primary antibodies against p-p38 mitogen-activated protein kinase (MAPK), p38 MAPK, p-JNK, c-Jun N-terminal kinase JNK, and *β*-actin were purchased from Abcam (Shanghai, China). They were detected with horseradish-peroxidase-conjugated secondary antibodies using the enhanced chemiluminescence (ECL) detection system. *β*-Actin was used as a loading control, and ImageJ software was used to analyze densitometry.

### 2.8. C. Elegans Strains and Treatment

*C. elegans* bristol N2 (wild-type) was provided by Dr. Du (Zhejiang University, China). The mutants were maintained at 20°C on a standard nematode growth medium (NGM) with *E. coli* OP50 as food resources. To collect eggs, adult animals on NGM were dissolved in bleaching solution. Then, the eggs were transferred into a new plate for hatching and synchronized at the same period.

Synchronized L3 stage *C. elegans* were collected and transferred to NGM containing THVF of different concentrations (2.5, 5, and 10 *μ*g/mL). After 24-hour treatments, 20 mM of glutamate was added while the concentration of TVE kept the same as before.

### 2.9. Determination of Survival Rate, Body Length, and Body Width

The survival rate assays were performed in the 24-well plates. A total of 40 young nematodes were raised in each well with culture medium (NaCl 3.1 g/L, KCl 2.4 g/L, cholesterol 1 mg/L), and *E. coli* OP50 was added for food supply. 0, 2.5, 5, and 10 *μ*g/mL THVF were added into medium, respectively. After treating for 24 h, 20 mM Glu was later added for another 24 h. Survival rate was defined as survival rate (%) = the living worm numbers after treatment/total worm numbers before treatment∗100%.

The survival rate in the control group was regarded as 100% when calculated. Meanwhile, the photos of *C. elegans* were captured by a microscope and animals' body length and body width were measured by Auto CAD.

### 2.10. Locomotion Behavior Assay

Head thrash and body bend assays were used to evaluate locomotion behavior ability of nematodes. Once, head thrash was defined as a change in the direction of bending in the mid body which was counted for 1 min. A body bend was defined as a change in the direction of the part of nematodes corresponding to the posterior bulb of the pharynx along the *y*-axis, assuming that the long axis of the body was the *x*-axis (*n* = 30 per replicate; three replicates per group).

### 2.11. Visualization of ROS, Superoxide, and GSH

To measure the level of ROS, superoxide, and GSH, three fluorescence probes (DCFE-DA, DHE, and NDA) were added, respectively. Images of animals were obtained through a fluorescence microscope.

### 2.12. Statistical Analysis

Data were expressed as mean ± standard deviations (SDs) from at least three independent experiments. Significant differences were determined by one-way analysis of variance (ANOVA) followed by the multiple comparison at *p* < 0.05. Significant differences were all analyzed using SPSS. Densitometry analyses were performed using Image-Pro Plus 6.0 software.

## 3. Results

### 3.1. The Main Compounds of THVF

**Figure 1:**
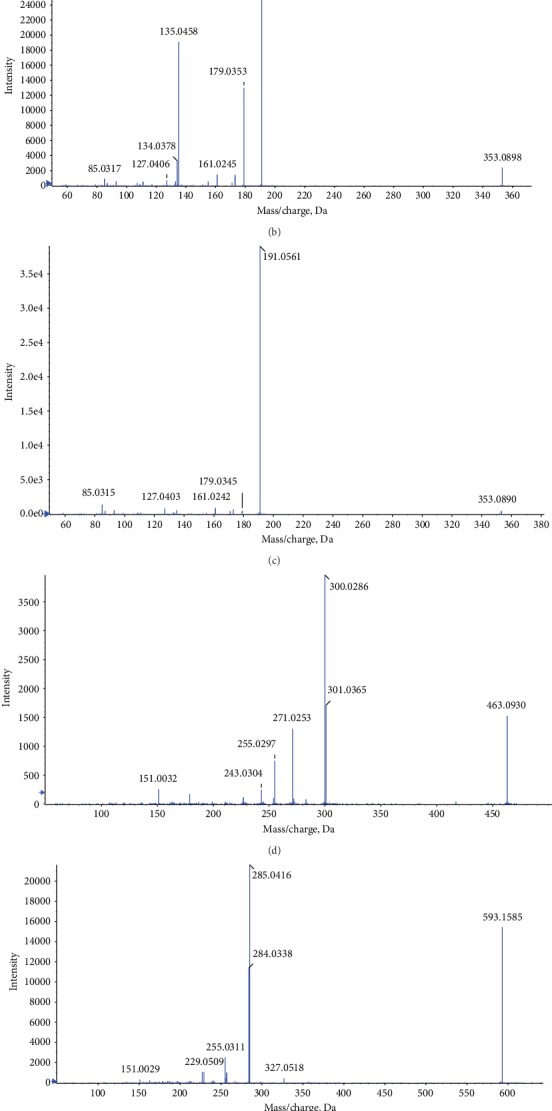
HPLC elution profile of compounds in THVF and effects of THVF on cytotoxicity in PC12 cells. (a) The liquid chromatography profile of THVF. The peak numbers were labeled according to the retention times. (b) MS/MS information for peak 1. (c) MS/MS information for peak 2. (d) MS/MS information for peak 3. (e) MS/MS information for peak 4. (f) PC12 cell viability was measured by the MTT method after treated with THVF at different concentrations for 24 h (*n* = 6).

UPLC-TOF/MS results showed that THVF was composed of four main compounds; we numbered these four compounds as peak 1, 2, 3, and 4 (Figure 1(a)). As Figure 1(b) illustrated, peak 1 with a molecular ion at *m*/*z* 191.0506 [quinic acid-H]^−^ was identified as C_16_H_18_O_9_ while a series of fragments of *m*/*z* 191.0561, 179.0345, 161.0242, 127.0403, and 85.0315 appeared in peak 2 secondary mass spectrum (Figure 1(c)). According to TOF/MS results and previous studies, peaks 1 and 2 were deduced as 3-caffeoylquinic acid and 5-caffeoylquinic acid [21, 25]. Peak 3 with a retention time at 33.923 min and fragment at *m*/*z* 301[M-H-146-162]^−^ was identified as quercetin-3-O-rutinoside. Furthermore, the relative molecular mass of peak 4 was 594, and a fragment of *m*/*z* 309 was lost at *m*/*z* 285 [M-H-308]^−^ that could correspond to the loss of one rutinoside and peak 7 could be kaempferol-3-O-rutinoside. Therefore, it could be deduced that THVF is consisted of 3-caffeoylquinic acid, 5-caffeoylquinic acid, quercetin-3-O-rutinoside, and kaempferol-3-O-rutinoside.

### 3.2. THVF Alleviated Oxidative Stress Caused by Glu

**Figure 2:**
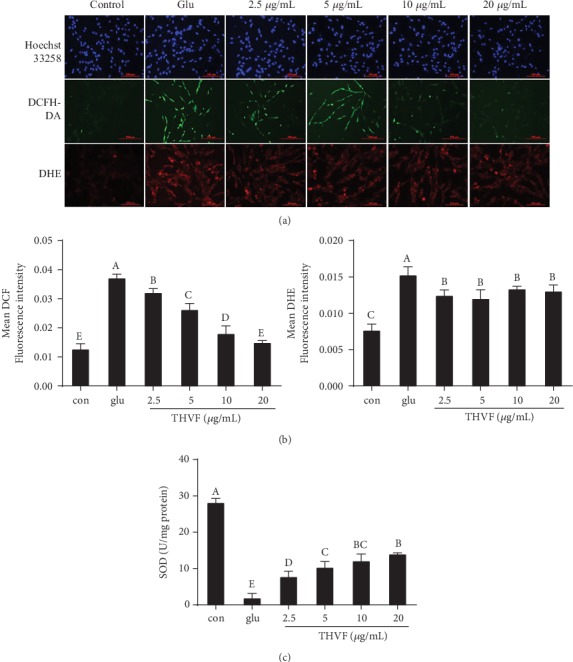
Effects of THVF on Glu-induced oxidative damage in PC12 cells (*n* = 3). (a) Hoechst 33258, DCFH-DA, DHE staining for genotoxicity, ROS, and O_2_^−^. (b) The quantitative data of panel DCFH-DA and DHE. (c) SOD activity of PC12 cells with or without Glu and THVF treatments. THVF-treated cells were inoculated in different concentrations of THVF for 24 hours, and then, 20 mM Glu was added for a total of 24 hours. Cells without Glu and THVF were used as negative control group. Cells treated with Glu alone were used as a glu group. Images were captured with a fluorescence microscope in the same settings. All the fluorescence images were quantified in the whole field with the background removed and represented by normalized fluorescence (*y*-axes) via Image-Pro Plus 6.0 (*n* = 3). Significance analysis was carried out according to the one-way ANOVA test, and different letters in figures mean statistically significant differences among the groups (a, b, c, etc., were labeled from large to small and once columns containing a same word means statistically insignificant, otherwise means statistically significant, *p* < 0.05).

According to the results of MTT assay (Figure 1(f)), we found that the concentration range of 0.78–25 *μ*g/mL showed no toxicity to PC12 cells. Thus, we choose 2.5, 5, 10, and 20 *μ*g/mL of THVF for further study. DNA damage is a key feature of cytotoxicity [26]. Therefore, Hoechst 33258, a specific DNA fluorescence probe, was adopted to assess nuclear fragmentation. As shown in Figure 2(a), the number of high light blue dots was markedly elevated after Glu stimulation while THVF treatments decreased such light dots at a dose-related manner.

Besides genotoxicity, Glu further caused intracellular redox disturbance, resulting in overproduction of ROS [27]. Since plant flavones were regarded as a potent free radical scavenger both *in vitro* and *in vivo* [28], we tested the intracellular ROS level in the presence or absence of THVF by DCFH-DA, a specific ROS fluorescence probe. An enhanced DCF fluorescence intensity was observed in Glu-treated cells compared with the control group (Figures 2(a) and 2(b)). Intriguingly, 10 and 20 *μ*g/mL THVF significantly declined the ROS level, with the DCF fluorescence intensity decreased to 0.18 and 0.15, respectively. Meanwhile, DHE, a unique probe of intracellular superoxide anion radicals, was further used to analyze O_2_^−^ contents. Similar findings were got, Glu significantly raised the mean DHE fluorescence intensity of PC12 cells (Figures 2(a) and 2(b)). In contrast, the intervention of THVF helped scavenging the overproduced O_2_^−^ with the DHE fluorescence intensity declined. Superoxide dismutase (SOD) is an antioxidant metalloenzyme that specifically acts as a disproportionation catalyst to suppress superoxide anion radical generation [29]. As Figure 2(c) demonstrated, Glu severely inhibited activity of SOD in PC12 cells. Fortunately, THVF reversed this situation and 20 *μ*g/mL THVF could even recover the SOD activity similar to control. These dates jointly revealed the protective effect of THVF under Glu-induced toxicity.

### 3.3. THVF Relieved Mitochondrial Dysfunction Induced by Glu

**Figure 3:**
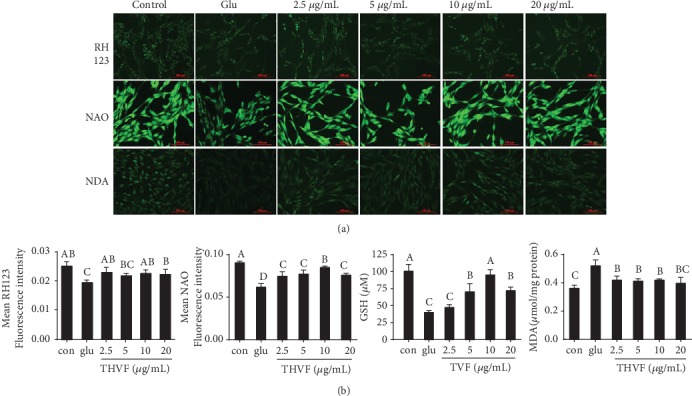
Effect of THVF on Glu-induced mitochondrial dysfunction in PC12 cells (*n* = 3). (a) Mitochondrial membrane potential, mitochondrial membrane lipid peroxidation, and GSH alterations of PC12 cells in the presence of Glu and THVF under different treatments and were incubated with RH123, NAO, and NDA probes. (b) The quantitative data of panel (a). Images were captured with a fluorescence microscope in the same settings. All the fluorescence images were quantified in the whole field with the background removed and represented by normalized fluorescence (*y*-axes) via Image-Pro Plus 6.0 (*n* = 3). Significance analysis was carried out according to one-way ANOVA test and different letters in figures mean statistically significant differences among the groups (a, b, c, etc., were labeled from large to small and once columns containing a same word means statistically insignificant, otherwise means statistically significant, *p* < 0.05).

ROS is often regarded as a by-product of mitochondrial dysfunction [30], and mitochondrial dysfunction could further accelerate the progression of various diseases, such as atherosclerosis, diabetes, Alzheimer's disease, and Parkinson's disease [31]. Based on these findings, we suspected that THVF might provide a defense effect to the toxicity via recovering mitochondrial function. RH123 fluorescence probe is specific for mitochondrial membrane potential (MMP) detection and NAO is for mitochondrial membrane lipid peroxidation (MMLP), respectively. As Figure 3(a) illustrated, a Rh123 fluorescence intensity decline could be observed with the stimulation of Glu, suggesting the decreased MMP, which is usually regarded as prerequisite and a landmark event in early apoptosis. Similarly, the NAO fluorescence intensity was markedly suppressed by Glu, indicating the disturbed MMLP. However, after THVF treatments, NAO intensity increased compared with Glu-treated cells. Malondialdehyde (MDA) is a crucial product of MMLP, and its production can aggravate damage and leading to aging and resistance physiology [32]. Consistent with previous results, Glu stimulation triggered MDA production and accumulation in PC12 cells while 2.5 *μ*g/mL was sufficient to decrease intracellular MDA content. Mitochondrial dysfunction could further facilitate consumption of glutathione (GSH), and NDA fluorescence intensity reduction was found after Glu stimulation. Conversely, THVF treatments increased intensity significantly, suggesting the alleviation of mitochondrial damage and enhancement of antioxidative ability. Based on these results, we believe THVF exerted a beneficial effect against Glu-induced mitochondrial dysfunction.

### 3.4. THVF Promoted Cell Proliferation Inhibited by Glu

**Figure 4:**
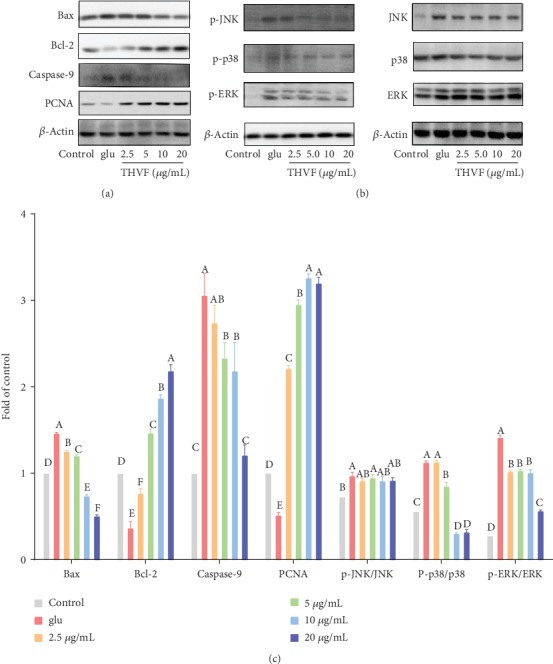
THVF treatment altered expressions of proteins in PC12 cells. (a) Western blot bands of Bax, Bcl-2, caspase-9, PCNA, and *β*-actin (*n* = 3). (b) Western blot bands of MAPKs. p-JNK, p-p38, and p-ERK represent phosphorylation of JNK, p38, and ERK. (c) Relative expressions of Bax, Bcl-2, caspase-9, PCNA, p-JNK, p-p38, p-ERK, JNK, p38, and ERK. The images were quantified by ImageJ software, the intensity of bands was corrected by *β*-actin (*n* = 3), and vertical lines in the histogram represent SDs of three replicates. Significance analysis was carried out according to the one-way ANOVA test, and different letters mean statistically significant differences among the groups (a, b, c, etc., were labeled from large to small and once columns containing a same word means statistically insignificant, otherwise means statistically significant, *p* < 0.05).

Besides genotoxicity, ROS generation, and mitochondrial dysfunction, Glu also directly induce PC12 cells apoptosis. B-cell lymphoma-2 (Bcl-2) protein family is closely related to apoptosis [33]. As Figure 4(a) showed, Glu upregulated the Bax expression and suppressed the protein level of Bcl-2, which manifested the apoptotic state of PC12 cells. Besides, the production level of caspase-9 was also increased to 3-fold of control. However, we found THVF obviously down-regulated expressions of Bax, caspase-9, and up-regulated Bcl-2 levels at a dose-dependent manner. PCNA plays a crucial role in cell proliferation and regulating cell cycle [34]. Consists with previous results, Glu significantly inhibited the expression of PCNA, indicating the damage of PC12 cell proliferation caused by Glu. On the contrary, THVF reversed such inhibition and up-regulated PCNA level to 2 times of control. These results suggested that THVF reversed apoptosis induced by Glu and facilitated cell proliferation.

### 3.5. The Protective Effect of THVF Involved in MAPK Pathways

As an important transmitter of signals from the cell surface to the interior of the nucleus, MAPKs can be activated by different extracellular stimuli such as cytokines, hormones, and cellular stress [35].  Figure 4(b) showed that with the stimulation of Glu, the phosphorylation of JNK, ERK, and p38 was upregulated. However, after THVF treatments, downregulation of phospho-p38 and phosphor-ERK was observed. Meanwhile, though THVF had both inhibited the expression level of total JNK and p-JNK, the ratio of p-JNK/JNK had not changed compared to Glu-induced cells. Based on these results, we deduced that THVF protect PC12 cells from toxicity via ERK/p38 pathways.

### 3.6. THVF Recovered the Locomotory Ability of *C. elegans*

**Figure 5:**
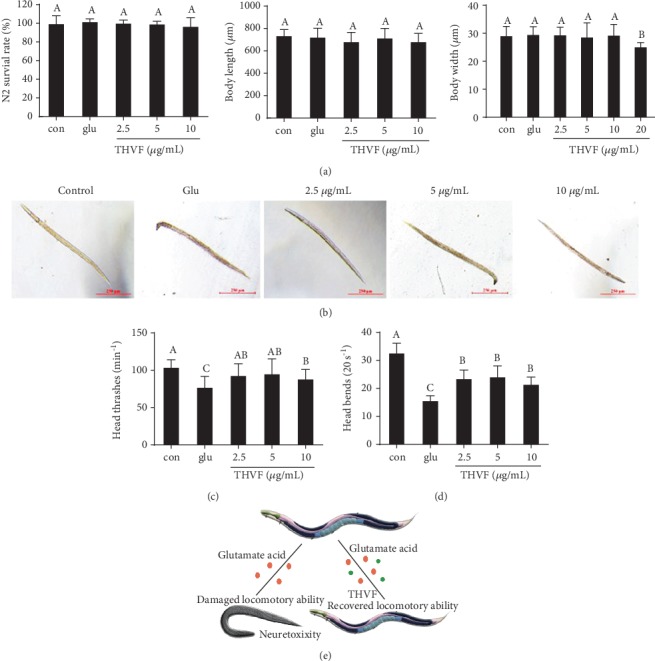
Effect of THVF on Glu-induced toxicity in *C. elegans* (*n* = 30). (a) Survival rate, body length, and body width of different groups in N2. (b) Representative photographs of *C.elegans* with different treatments. (c) Head thrashes of different groups in N2. (d) Body-bending frequency of different groups in N2. (e) Scheme illustration of Glu and THVF on nematode locomotory activity. Significance analysis was carried out according to the one-way ANOVA test, and different letters show statistically significant differences among the groups (*p* < 0.05).

With short lifespan and low cost, *C. elegans* is regarded as an ideal *in vivo* model to study toxicity [36]. As shown in Figures 5(a) and 5(b), both Glu and THVF had not affected the survival rate, body length, and body width of nematode. However, Glu stimulation caused severe damage to nematodes' locomotory ability. Our results showed significant decreases in body bends and head thrashes of *C elegans* after exposure to Glu (Figures 5(c) and 5(d)), implying the neurotoxicity of Glu, while THVF partly recovered the locomotory abilities of nematodes. Based on these results, our results demonstrated that Glu could induce considerably severe locomotor defects and THVF was capable to protect nematodes from Glu-induced neurotoxicity (Figure 5(e)).

### 3.7. THVF Protected *C. elegans* from Glu-Induced Oxidative Stress

**Figure 6:**
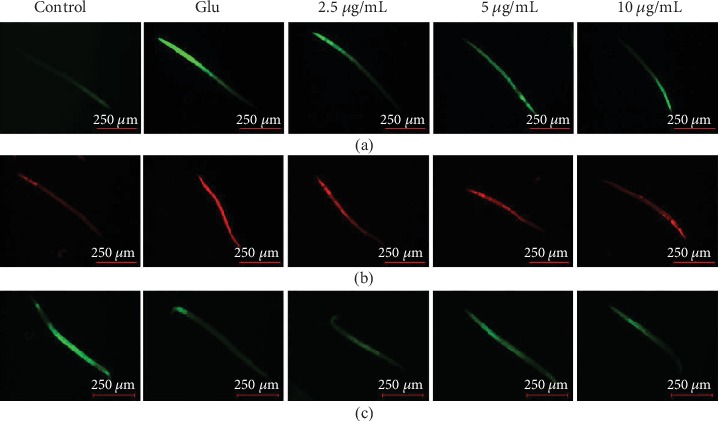
Fluorescence staining for *C. elegans* (*n* = 30). (a) DCFH-DA staining for ROS alteration. (b) DHE staining for O_2_^−^alteration. (c) NDA staining for GSH contents. Images were captured with a fluorescence microscope in the same settings (*n* = 6).

In *in vitro* study, Glu induced oxidative toxicity to PC12 cells and THVF effectively alleviated oxidative stress. To confirm whether THVF could help prevent from oxidative damage *in vivo*, we used DCF, DHE, and NDA probes to measure intracellular ROS, O_2_^−^, and GSH depletion in *C. elegans*, respectively.  Figure 6 demonstrated that the treatment of Glu remarkably increased the mean DCF fluorescence intensity whereas the addition of THVF restored the mean DCF fluorescence intensity as expected at a dose-related manner. Moreover, Glu enhanced the level of O_2_^−^ and facilitated the GSH depletion in nematodes. On the contrary, THVF intervention suppressed the generation of O_2_^−^ and restrained GSH depletion. These results above suggested that THVF could attenuate Glu-induced oxidative damage in *C. elegans*.

## 4. Discussion

The Chinese have used certain seaweeds to enhance the flavor of food for some 2,000 years. In 1908, the flavor-enhancing agent was identified as glutamic acid [37]. Nowadays, Glu has been applied to a large amount of industrial production with its various applications such as flavor enhancer, wrinkles reducer, energy supplier, and neuro-therapeutic agent. However, the toxicity especially neuro-toxicity of Glu has been continuously uncovered recently. Many studies have revealed that natural products are able to help alleviate toxicity and protect from outer stimulation. TH is a traditional Chinese herb and food with various confirmed bioactive functions, while TH vine is usually regarded useless and often discarded as a by-product, resulting in a waste of source. We extracted and purified the flavones of TH and use UPLC-TOF/MS to characterized the main compounds of THVF. Results showed that THVF was composed of 3-caffeoylquinic acid, 5-caffeoylquinic acid, quercetin-3-O-rutinoside, and kaempferol-3-O-rutinoside.

With extreme versatility for pharmacological manipulation, ease of culture, and the large amount of background knowledge on their proliferation and neuro characterization, rat phaeochromocytoma (PC12) cells have been widely used as a model for neural research [38]. Glu caused severe genotoxicity to PC12, hereby triggered enhanced ROS generation and overproduction of O_2_^−^, which were by-products of mitochondrial dysfunction induced by Glu. Additionally, Glu inhibited the SOD enzyme activity and then increased the MDA accumulation as results. Conversely, THVF attenuated the Glu-induced toxicity by alleviating the genotoxicity, relieving oxidative stress, and recovering mitochondrial functions. It is implying that cell death in central nervous system irregulating may play a part in an etiology of cancer, AIDS, autoimmune diseases, and degenerative diseases such as Alzheimer's disease (AD), amyotrophic lateral sclerosis (ALS), and Huntington's disease. Similarly, PC12 cell proliferation was inhibited by Glu with down-regulation of PCNA and Bcl-2, as well as up-regulation of Bax and caspase-9, suggesting the apoptosis of cells. As expected, THVF recovered the cell proliferate ability and enhanced the expression levels of PCNA, Bcl-2.

Mitogen-activated protein kinase (MAPK), as an important transmitter of signal transmission from the cell surface to the interior of the nucleus, plays a key role in stress response [35]. Glu markedly activated the phosphorylation of p38, JNK, and ERK. Nevertheless, down-regulation of p38 phosphorylation was found in THVF treated cells. Considering p38 MAPK is vital in immune response to stress and cell survival [39], THVF can inactivate, at least partly, the p38 MAPK pathway. Besides, THVF suppressed the expression of Glu-induced ERK over-phosphorylation. Previous studies have reported that ERKs, as a downstream protein of various growth factors (EGF, NGF, PDGF), regulate cell proliferation, differentiation, and survival. It acts like a receptor of signals from growth factors, mitogens, and environmental stimuli and then regulates nuclear transcription factors through the ERK signal cascade [40]. Thus, THVF might help protect PC12 cells from Glu-induced toxicity by suppressing over-phosphorylation of ERK and p38.

It has been proved that neurotransmitters and their metabolism, vesicle circulation, and synaptic transmission are highly conserved, and all 302 neurons of the nematode have been well studied, which makes *C. elegans* an ideal model to learn neurotoxicity and behavior *in vivo* [41]. Locomotor behaviors require the control of neural circuits [42]. Our results implied that Glu toxicity might be involved in the disruption of motor control function or appropriate synaptic contacts between neurons and muscle cells. As *C. elegans* lacks afunctional blood-brain barrier, Glu could quickly diffuse into the nervous system and directly produce neurotoxic actions, resulting in damaged head thrashes and body bends. Fortunately, here THVF turned to be a neuroprotective agent and recovered the locomotory ability of nematodes. Consistent with *in vitro* results, Glu elevated the intracellular ROS, O_2_^−^ generation, and GSH depletion in nematodes and THVF prevented larvae from Glu-toxicity. However, it is unclear whether MAPK pathways are also involved in the THVF-treated nematodes, and further research is needed to confirm the inner mechanism in *C. elegans* level.

## 5. Conclusion

In this study, we focus on Glu neurotoxicity rather than its wide applications and well-known protective effects. As results illustrated, Glu caused damage to PC12 cells and *C. elegans* while THVF, flavones extracted, and purified from TH vine was able to protect Glu-induced toxicity via MAPK pathways. These data provide a novel insight and raise worthwhile questions about the Glu-accompanied side-toxicity and THVF potential neuroprotective effects both *in vitro* and *in vivo*, as well as MAPK pathways' role in neurotoxicity.

## Data Availability

The data used to support the findings of this study are available from the corresponding author upon request.
